# Association Analysis Between Genotype and Environment: Differentiation Between *Cyclocarya paliurus* Resources That Accumulate Triterpenoids

**DOI:** 10.3389/fpls.2022.945897

**Published:** 2022-08-05

**Authors:** Caowen Sun, Xulan Shang, Shengzuo Fang, Wanxia Yang, Yanni Cao, Haifen Ding, Xiaochun Li

**Affiliations:** ^1^College of Forestry, Nanjing Forestry University, Nanjing, China; ^2^Co-Innovation Center for Sustainable Forestry in Southern China, Nanjing Forestry University, Nanjing, China

**Keywords:** triterpenoids, genotype, genetic resource, *Cyclocarya paliurus*, association analysis

## Abstract

*Cyclocarya paliurus* is mainly distributed in subtropical areas of China. Its leaves are rich in beneficial triterpenoids that have bioactivities against human diseases, including hyperlipemia, diabetes, and hypertension. In this study, data on the genetic diversity, distributing environment, and triterpenoids of *C. paliurus* samples were collected from a wide area in China. The data covered 316 *C. paliurus* germplasms collected from 26 distinct populations. Association analysis between genotype and triterpenoids was carried out to describe triterpenoids accumulation pattern. Based on our analyses, we identified the important trend that genotypes with higher triterpenoid contents belonged to a unique genotype subgroup. The results showed that pterocaryoside B and pterocaryoside A significantly vary among the genotypic subgroups. In addition, the different genotypic subgroups showed distinct geographical distributing areas. These findings provide information about the relationship between genetic and environmental factors and how this affects triterpenoids accumulation. This information will be valuable for targeted breeding and for further germplasm selection of *C. paliurus* resources.

## Key Message

An association analysis based on genotypic, biochemical, geographical, and environmental data revealed that there are high-triterpenoid *Cyclocarya paliurus* subgroups. Each subgroup responds differently to environmental and climatic factors.

## Introduction

*Cyclocarya paliurus* is the only extant species in the *Cyclocarya* genus, which is distributed only in subtropical areas of China (Manchester et al., 2009; Sun et al., 2021). The leaves are rich in triterpenoids, which can improve insulin resistance and metabolism disorder of glucose and lipid, and have the effects of reducing the lipids and pressure of blood (Kurihara et al., 2003; Xie et al., 2006). However, the contents of triterpenoids in leaves of *C. paliurus* vary greatly, which hindrances the development of the *C. paliurus* industry. Previous studies have analyzed the composition of compounds in leaves of *C. paliurus*, including six main triterpene compounds (e.g., arjunolic acid, cyclocaric acid B, pterocaryoside B, pterocaryoside A, hederagenin, and oleanolic acid) (Shu et al., 1995; Wright et al., 2014; Shang et al., 2015). While the triterpenoids variation patterns of different *C. paliurus* genotypes remain unclear.

Currently, considerable efforts are focused on the selection of *C. paliurus* resources according to their patterns of secondary metabolite accumulation (Cao et al., 2017; Liu et al., 2018; Sun et al., 2020a,b). Previous studies have estimated the genetic diversity of *C. paliurus* populations (Li X. C. et al., 2017; Qu et al., 2021a,b), the responses of secondary metabolites to cultivation measures (Deng et al., 2015, 2018; Yang et al., 2017; Liu et al., 2018; Wang et al., 2021), and secondary metabolites variations among populations (Fang et al., 2011; Cao et al., 2017, 2018). Genotyping tests of the *C. paliurus* germplasm have been carried out at the population level, and have shown that different genotypes respond differently to abiotic factors (Deng et al., 2015, 2017, 2019; Liu et al., 2016). In previous studies, variations in secondary metabolite accumulation could only be attributed to the specific genotype being studied, and no general patterns were identified. Cao et al. (2018) noted this and considered that there were huge variations in secondary metabolite accumulation both at the population level and at the individual level. Thus, the degree to which the genotype explains variations in secondary metabolite accumulation and the balance among different metabolites is unknown. Clarifying these relationships may be the key to understanding the strong effect of the genotype on the quality of *C. paliurus* resources, especially when it is hard to construct clonal propagation systems.

In this research, based on previous studies on the genotype, geographic variation, and biochemistry of *C. paliurus*, data from multiple *C. paliurus* germplasms were collected and analyzed to investigate the patterns of triterpenoids accumulation. The objectives were to identify the effects of genotype on the triterpenoids accumulation in *C. paliurus*. Based on our analyses, we tried to identify general patterns of triterpenoids accumulation at the genotype level. These results will be valuable for targeted breeding and for further germplasm selection.

## Materials and Methods

### Plant Materials

Leaves were collected from *C. paliurus* trees in China in 2014 (Li X. C. et al., 2017; Cao et al., 2018). The leaves were collected from 316 individual trees at 26 different sites (Table 1). The sites were distributed across 12 provinces in China, covering a range of 5.4° latitude, 12.6° longitude, and 1,370 m elevation (Table 1). Mature leaves were collected in October to ensure that leaf secondary metabolites were fully developed. The leaves were mainly from dominant or subdominant branches of trees that were >20 years old. All samples were dried, sliced, ground, and stored at room temperature until analysis.

**Table 1:** Collection locations and associated environmental factors.

**Population code**	**Location**	**Latitude (**°**N)**	**Longitude (**°**E)**	**Altitude (m)**	**Annual mean temperature (**°**C)**	**Annual mean rainfall (mm)**	**Annual sunlight (h)**	**Frost-free days (day)**	**Sample size**
JQ6	Jixi County, Anhui	30.15	118.89	730	12.1	1,726	1,920	233	11
QJ4	Jingde County, Anhui	30.23	118.45	610	13.8	1,657	1784.1	240	3
QJ4	Qimen County, Anhui	30.02	117.53	400	13.9	1,775	1,669	232	5
SW5	Shucheng County, Anhui	31.02	116.54	770	12.3	1,606	1,969	224	12
FS13	Ningbo City, Zhejiang	29.76	121.22	715	14	1,527	1,850	232	8
LF14	Longquan City, Zhejiang	27.91	119.19	1,200	11.8	2,119	1849.8	263	7
LF14	Wencheng County, Zhejiang	27.88	119.79	915	13.9	1,913	1764.4	285	5
PK11	Pucheng County, Fujian	27.93	118.76	930	15.8	1,998	1,900	254	10
SM12	Mingxi County, Fujian	26.57	116.93	564	17.4	1,821	1788.6	261	12
H29	Lueyang County, Shanxi	33.36	105.87	1,200	13	714	1558.3	236	9
JY2	Yongshun County, Hunan1	28.88	110.33	670	14.8	1,492	1,306	286	9
SS1	Shaoyang City, Hunan	26.37	110.13	1,000	16.7	1,320	1348.9	304	16
JY3	Yongshun County, Hunan2	24.92	112.03	845	17.4	1,502	1,758	308	10
NH22	Hefeng County, Hubei	29.88	110.42	1,125	11.3	1,499	1,342	245	6
YW21	Wufeng County, Hubei	30.19	110.9	969	12.6	1,650	1,533	240	15
JX17	Xiushui County, Jiangxi	28.16	114.52	827	13.5	1,655	1600.4	247	15
JJ18	Jinggangshan County, Jiangxi	26.51	114.1	967	13.2	1,816	1,511	241	10
FD16	Fenyi County, Jiangxi	27.63	114.53	565	15.3	1,691	1,251	270	14
BL23	Baise City, Guangxi1	24.46	106.34	1,448	15.6	1,364	1906.6	357	12
BT24	Baise City, Guangxi2	24.61	104.95	1,770	16.7	1,212	1569.3	357	11
GL25	Longsheng County, Guangxi	25.62	109.89	606	14.5	1,629	1,309	314	11
GZ26	Ziyuan County, Guangxi	25.92	110.38	850	15.5	1,580	1,275	300	10
QL9	Liping County, Guizhou	26.34	109.24	727	16.1	1,311	1317.9	277	11
QJ10	Jianhe County, Guizhou	26.37	108.38	1,240	15.1	1,265	1236.3	300	15
TS7	Shiqian County, Guizhou	27.35	108.11	1,239	13.3	1,256	1232.9	316	16
TL8	Yinjiang County, Guizhou	27.74	108.51	1,032	14.5	1,231	1,255	300	7
M20	Muchuan County, Sichuan	28.97	103.78	1,200	17.3	1,332	968	332	34
GQ19	Guangyuan City, Sichuan	32.42	104.86	1,570	14.1	1,027	1,292	243	12

### Determination of Triterpenoids

**Figure A1:**
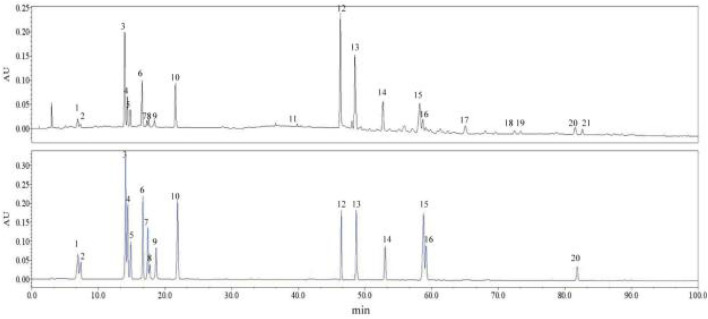
HPLC chromatograms of a representative sample solution (top) and a mixed standard solution containing all quantitative compounds (bottom).

Triterpenoids were identified and quantified in our previous studies (Cao et al., 2017, 2018). Briefly, powdered leaves (75 g from each location) were extracted with 80% ethanol in a water bath at 90°C for 2 h, then shaken for 15 min. The mixture was centrifuged at 8,000 r/min for 15 min, and a cooling centrifuge Sigma 3-18KS instrument was applied in the present study (3-18KS; Sigma, Aachen, Germany). Supernatants were concentrated at 40°C to obtain extracts. Triterpenoid contents were analyzed by high-performance liquid chromatography (HPLC) according to Cao et al. (2017, 2018), with a C18 column (X-Bridge, 250 × 4.6 mm; Waters, Milford, MA, USA) connected to an Agilent 1200 series HPLC system (Agilent, Waldbronn, Germany). A stepwise elution program was applied according to Cao et al. (2017, 2018). The characteristic common peaks of individual triterpenoids, which were sufficient to evaluate the quality of *C. paliurus* leaves, were identified and quantified by comparison with external standards (Liu et al., 2018). The reference standards of hederagenin, arjunolic acid, and oleanolic acid (purity >98%) were purchased from Shanghai Yuanye Biotechnology Co., Ltd. (Shanghai, China), and cyclocaric acid B, pterocaryoside A, and pterocaryoside B (purity >98%) were isolated and purified from the laboratory of China Pharmaceutical University (Nanjing, China) (Cao et al., 2017) (Figure A1; Table A1).

### Environmental Factors

Geographical data (longitude, latitude, and altitude) were collected at each survey site with a GPS unit (JUNO® SCSD, Trimble, Sunnyvale, CA, USA). Climate data were obtained from ClimateAP for the historical period 1991–2014 according to Liu et al. (2018) and included annual mean temperature, annual mean rainfall, annual sunlight, and the number of frost-free days in each region (http://climateap.net/).

### Extraction of Genomic DNA and PCR Amplification

The DNA extraction and PCR amplification were conducted in a previous study (Li X. C. et al., 2017) (Table 2). Briefly, DNA was isolated from dried leaves and purified using the Ezup Column Plant Genomic DNA Purification Kit (Sangon Biotech, Shanghai, China). Nine inter simple sequence repeat (ISSR) and 6 simple sequence repeat (SSR) selected primers were selected based on the reproducibility and band polymorphism (Li X. C. et al., 2017). The products were separated by 8% non-denatured polyacrylamide gel electrophoresis (PAGE) and silver-stained. The genetic similarity among populations was assessed by PCA analysis by Gen Al Ex V.6.5 Mxcomp.

**Table 2:** Triterpenoid concentrations in leaves of 316 *C. paliurus* individuals.

**Triterpenoids**	**Minimum (mg/g)**	**Maximum (mg/g)**	**Variable coefficient**	**Median (Quartile) (mg/g)**
Arjunolic acid	0.19	11.47	0.60	2.97 (1.94–4.42)
Cyclocaric acid B	0.29	6.4	0.70	1.25 (0.77–1.94)
Pterocaryoside B	0	19.17	1.08	1.97 (0.41–4.02)
Pterocaryoside A	0.12	16.41	0.75	3.04 (1.68–4.75)
Hederagenin	0.02	4.68	0.71	0.84 (0.55–1.32)
Oleanolic acid	0.07	1.51	0.62	0.35 (0.23–0.49)
Content of total six triterpenoids	1.68	44.5	0.56	11.39 (7.51–16.24)

**Table 3:** Results of PCA analysis of triterpenoids of *C. paliurus*.

	**Extract coefficient**	**PCA1**	**PCA2**
Arjunolic acid	0.69	0.81	−0.18
Cyclocaric acid B	0.72	0.76	−0.37
Pterocaryoside B	0.90	0.47	0.82
Pterocaryoside A	0.82	0.81	0.40
Hederagenin	0.63	0.69	−0.39
Oleanolic acid	0.68	0.68	−0.47
Content of total six triterpenoids	1.00	0.94	0.33
Total variance of interpretation (%)		56.39	21.24

**Table 4:** *C. paliurus g*enotype structure differentiation (Li X. C. et al., 2017).

**Genotype group**	**Location (sample size)**	**No. of samples**
1	Guangyuan City, Sichuan (12)	12
2	Ningbo City, Zhejiang (8), Longquan City, Zhejiang (7), Pucheng County, Fujian (10), Mingxi County, Fujian (12), Xiushui County, Jiangxi (15), Jinggangshan County, Jiangxi (10), Fenyi County, Jiangxi (14)	76
3	Jixi County, Anhui (11), Jingde County, Anhui (3), Qimen County, Anhui (5), Shucheng County, Anhui (12), Wencheng County, Zhejiang (5), Lueyang County, Shanxi (9), Yongshun County, Hunan1 (9), Shaoyang City, Hunan (16), Yongshun County, Hunan2 (10), Hefeng County, Hubei (6), Wufeng County, Hubei (15), Baise City, Guangxi1 (12), Baise City, Guangxi2 (11), Longsheng County, Guangxi (11), Ziyuan County, Guangxi (10), Liping County, Guizhou (11), Jianhe County, Guizhou (15), Shiqian County, Guizhou (16), Yinjiang County, Guizhou (7), Muchuan County, Sichuan (34)	228

### Association Analyses

We combined the genetic diversity data with triterpenoids data and environmental data for the association analysis. The secondary metabolite data concluded triterpenoids compounds in 316 individuals. The genetic diversity data were for 26 populations at the population level. The environmental data obtained as described above (sections Environmental Factors and 2.4) were collected at the population level.

A principal component analysis (PCA) was constructed to describe the accumulation characteristics of triterpenoids. According to the genotype division, another PCA was conducted to detect differences in patterns of genotypic subgroups distributing characteristics related to environmental variables. Secondary metabolite data were marked with specific genotypic subgroups for the association analysis between genotype and secondary metabolite contents. Multiple comparisons, non-parametric tests, and PCA were conducted using SPSS software, version 19.0.0 (SPSS Inc., Chicago, IL, USA).

## Results

### Triterpenoid Accumulation in *C. paliurus* Leaves

The *C. paliurus* leaves were collected from 316 individual trees of 26 different population (Table 2), and the total triterpenoid content ranged from 1.68 to 44.50 mg/g (the median, 11.39 mg/g) and was the sum of six compounds: arjunolic acid (2.97 mg/g), pterocaryoside B (1.25 mg/g), pterocaryoside A (1.97 mg/g), cyclocaric acid B (3.04 mg/g), hederagenin (0.84 mg/g), and oleanolic acid (0.35 mg/g). Among them, the variable coefficient ranged from 0.56 to 1.08, cyclocaric acid B (0.70), pterocaryoside B (1.08), pterocaryoside A (0.75), and hederagenin (0.71) exhibit higher variable coefficient, while arjunolic acid (0.60), oleanolic acid (0.62), and total six triterpenoids (0.56) exhibit lower.

**Figure 1:**
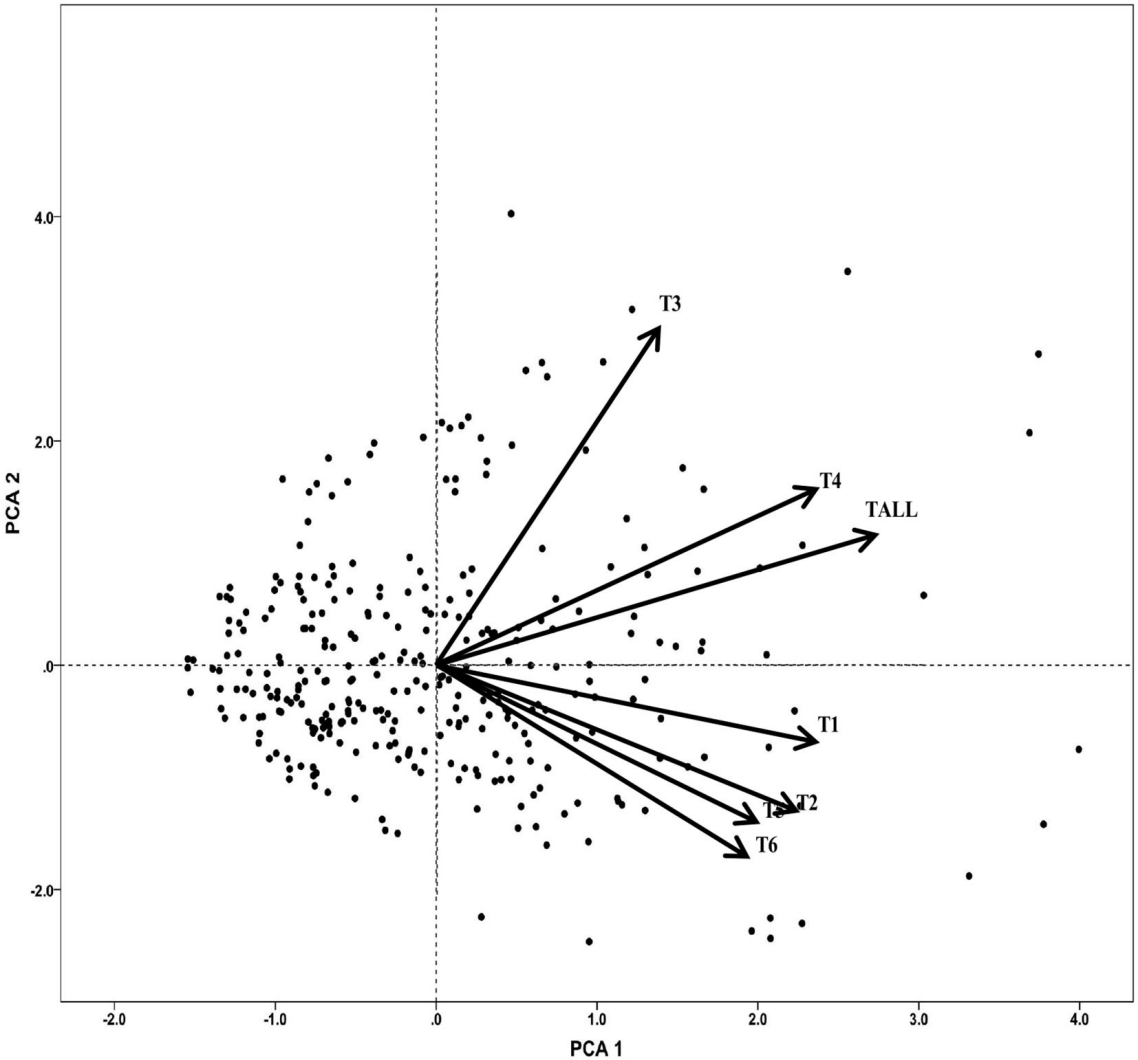
Principal component analysis of triterpenoids of *C. paliurus* germplasm. T1, arjunolic acid; T2, cyclocaric acid B; T3, pterocaryoside B; T4, pterocaryoside A; T5, hederagenin; T6, oleanolic acid; Tall, total triterpenoid.

To explore the triterpenoids variation pattern, a principal component analysis of 7 triterpenoid indicators was constructed (Figure 1). In general, the first principal components have explained 77.6% of total variance with PCA 1 explaining 56.39% and PCA 2 explaining 21.24%. Most triterpenoid information was included, especially for total triterpenoid (1), cyclocaric acid B (0.72), pterocaryoside B (0.90), and pterocaryoside A (0.82) (Table 1). As shown in Figure 1, different types of triterpenoids showed almost the same variation direction, indicating that they changed in basically the same pattern.

### Genetic Structure of Natural Populations

In a previous study on the genetic diversity of *C. paliurus* resources, no significant differences in coefficients were detected in a UPGMA (Li X. C. et al., 2017). Despite long-term geographical isolation, the 26 populations separated into five groups at the 0.91 level in the ISSR and SSR marker-based UPGMA dendrogram: GQ19, TS7, and SS1 each formed separate groups (Li X. C. et al., 2017); group 1 consisted of PK11, SM12, LF14, FS13, FD16, JX17, and JJ18; and group 2 consisted of JY2, M20, H29, YW21, NH22, QJ4, SW5, JQ6, QL9, TL8, QJ10, JY3, BL23, BT24, GL25, and GZ26. However, the UPGMA method seemed to produce ambiguous results that were rather limited for association analyses of population division.

**Figure 2:**
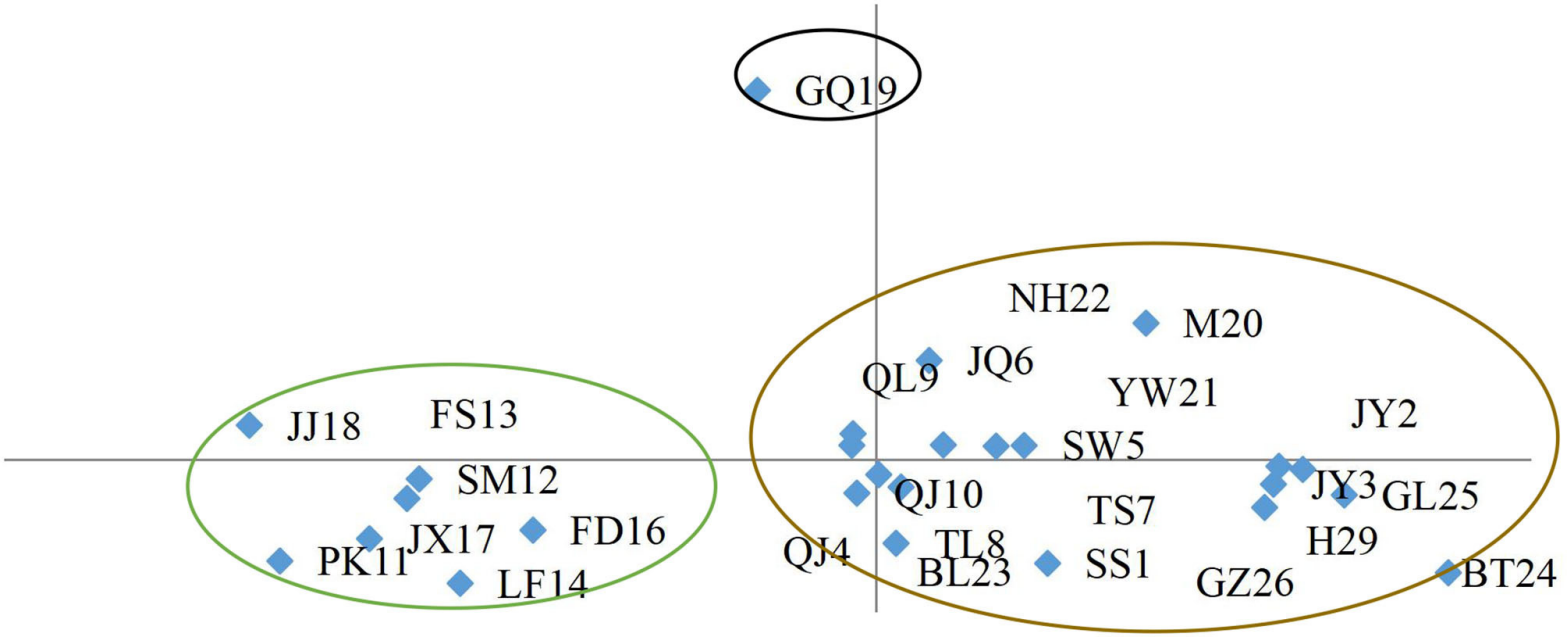
PCA grouping *C. paliurus* populations based on ISSR and SSR marker data.

In this study, instead of conducting analyses at the population or individual levels as was done in previous studies (Deng et al., 2015, 2017, 2019; Liu et al., 2016, 2018; Cao et al., 2017, 2018; Zhou et al., 2019), we conducted a PCA to analyze the phylogenetic relationships (Li X. C. et al., 2017) in the *C. paliurus* population (Figure 2). The 26 populations were divided into three clear subgroups according to the first two components. Subgroup I included population GQ19, subgroup II included populations JJ18, FS13, SM12, JX17, PK11, FD16, and LF14, and subgroup III contained the populations NH22, M20, QL9, JQ6, YW21, SW5, JY2, QJ10, TS7, JY3, GL25, QJ4, TL8, BL23, SS1, GZ26, H29, and BT24. This partly agreed with the results of the previous UPGMA clustering analysis (Li X. C. et al., 2017), but the groups were more clearly defined for further analyses.

According to the PCA results, 26 different *C. paliurus* population has been divided into three genotype subgroups. The first genotype subgroup included 12 individuals of the *C. paliurus* population located in Guangyuan City, Sichuan. And the second genotype subgroup included 76 individuals of 7 *C. paliurus* populations from Zhejiang province, Fujian province, and Jiangxi province. The third genotype subgroup included 228 individuals of 20 *C. paliurus* populations from Anhui province, Zhejiang province, Shanxi province, Hunan province, Hubei province, Guangxi province, Guizhou province, and Sichuan province. According to the clustering result, most germplasm resources belong to genotype subgroup 3, and subgroup 1 only contained 1 population.

### Genotype and Environment Association Analysis

**Figure 3:**
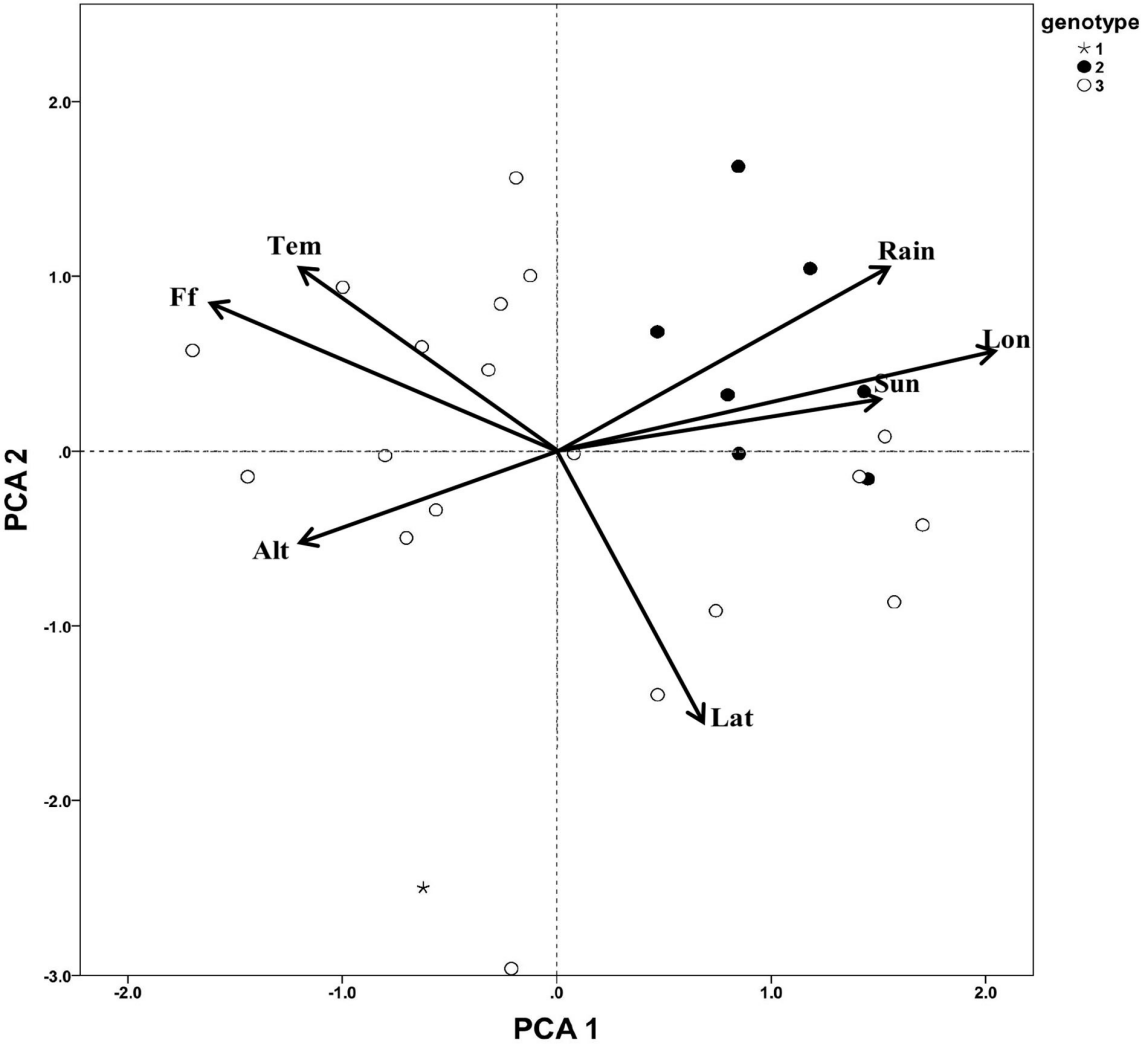
Principal component analysis of distributing environment factors of *C. paliurus* germplasm. Tem, annual mean temperature; Rain, annual precipitation; Lat, latitude; Lon, Longitude; Sun, annual sunlight hour; Alt, altitude; Ff, frost-free days.

To determine whether the different genotype subgroups of *C. paliurus* showed different responses to environmental factors, a PCA was carried out to analyze the distributing environmental data of different genotype *C. paliurus* subgroups (Figure 3). The first two PCAs (Table 5), analyzed 7 environmental variables, namely, latitude, longitude, altitude, annual mean temperature, annual mean rainfall, annual sunlight, and frost-free days, explained 74.95% of the total variation (PC 1 explained 44.95% and PC 2 explained 30%). Most environmental information was included, especially for latitude (0.83), longitude (0.95), annual mean rainfall (0.80), and frost-free days (0.90). According to the results, the three genotype subgroups showed a significant difference in geographical distributing patterns. As Figure 3 showed, individuals from genotype subgroup 2 were almost all distributed in the first quartile, with higher longitude, annual mean rainfall, and annual sunlight. While individuals from genotype subgroup 3 were almost all distributed in the second and fourth quartile, with higher latitude, annual mean temperature, and frost-free days. And genotype subgroup 1 was distributed only in the third quartile with higher altitudes.

**Table 5:** Results of PCA analysis of environmental factors of *C. paliurus*.

	**Extract coefficient**	**PCA1**	**PCA2**
Latitude	0.83	0.34	−0.85
Longitude	0.95	0.93	0.28
Altitude	0.58	−0.67	−0.35
Annual mean temperature	0.70	−0.62	0.56
Annual precipitation	0.80	0.72	0.54
Annual sunlight	0.50	0.69	0.15
Annual frost-free days	0.90	−0.86	0.41
Total variance of interpretation (%)		44.95	30.00

To further analyze the geographical variation pattern, a Kruskal–Wallis test was made to find the environment factors with significant differences between the three genotype subgroups (Table 6). According to the result, it was found that the geographical distribution of different genotype subgroups exhibits significant difference along with longitude (*P* < 0.05) and annual mean rainfall (*P* < 0.01). Multiple comparison results also showed that genotype subgroup 2 was almost distributed in the environment with the median longitude being 114.53° (quartile 114.52°-118.76°), and genotype subgroup 3 almost distributed at lower longitude being 109.24° (quartile 106.34°-110.9°). Besides, genotype subgroup 2 is almost all distributed in the environment with the median annual mean rainfall being 1,816 mm (quartile 1,655–1,821 mm), much higher than genotype subgroup 3 with the median being 1,332 mm and the quartile being 1,265–1,606 mm. In general, environment gradient, including longitude and annual mean rainfall, has been certified significantly correlated with the genotype geographical differentiation of *C. paliurus* germplasm.

**Table 6:** Kruskal–Wallis test and multiple comparisons between genotypes and environmental factors of *C. paliurus*.

**Environment factors**	**Significance**	**Genotype 1**	**Genotype 2**	**Genotype 3**
Latitude	0.347			
Longitude	0.011	104.86Aa	114.53 (114.52–118.76)Cc	109.24 (106.34–110.9)Bb
Altitude	0.189			
Annual mean temperature	0.997			
Annual mean rainfall	0.001	1027Aa	1,816 (1,655–1,821)Cc	1,332 (1,265–1,606)Bb
Annual sunlight	0.429			
Frost-free days	0.548			

*Lower case letters indicate that the environmental factors have a significant difference at the P < 0.05 level in genotypes; upper case letters indicate that different genotypes have a significant difference at the P < 0.01 level*.

### Genotype Subgroups Associated With Particular Patterns of Triterpenoids Accumulation

**Figure 4:**
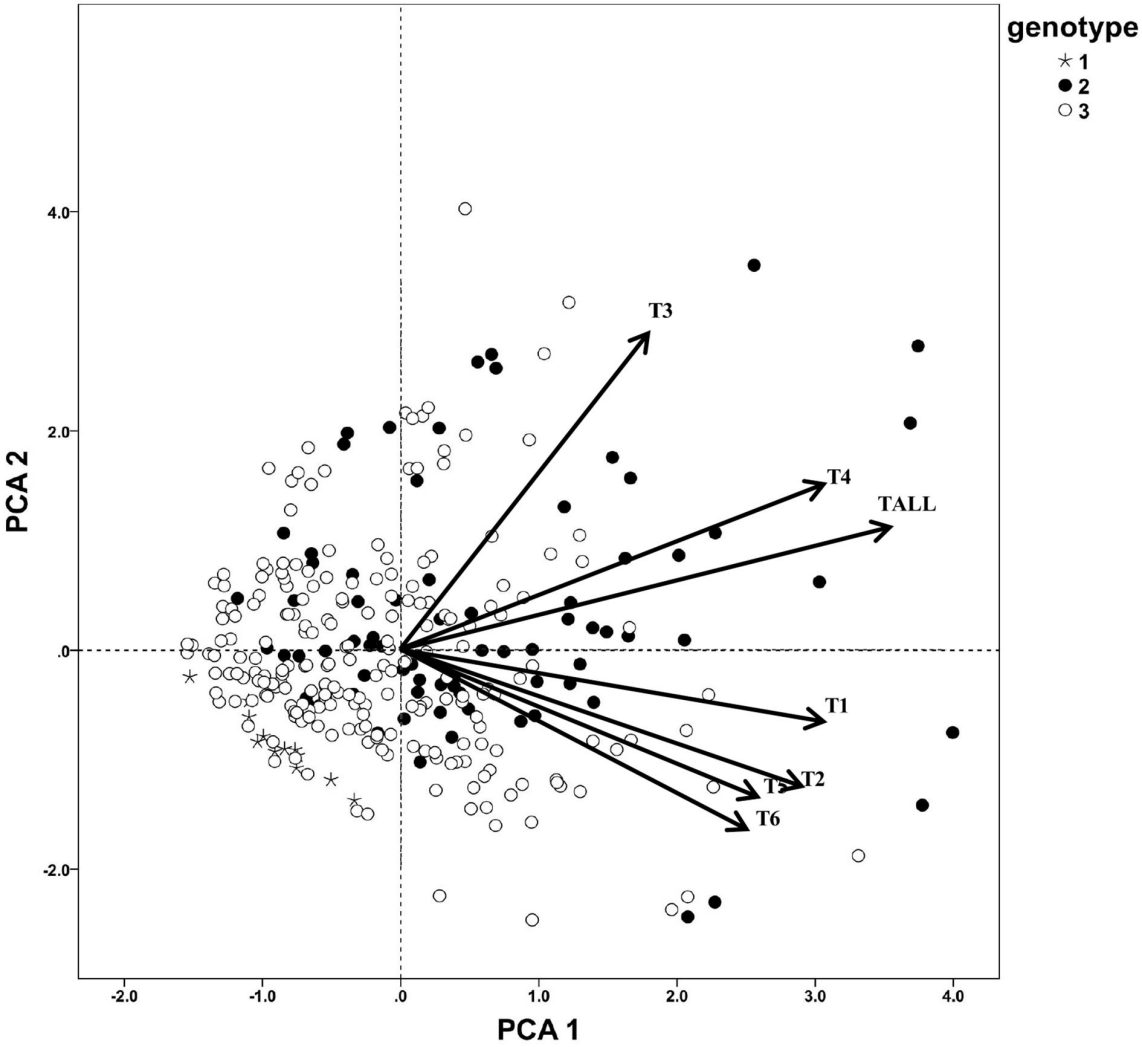
Principal component analysis of triterpenoids of *C. paliurus* genotypes. T1, arjunolic acid; T2, cyclocaric acid B; T3, pterocaryoside B; T4, pterocaryoside A; T5, hederagenin; T6, oleanolic acid; Tall, total triterpenoid.

According to the secondary triterpenoids variation pattern of *C. paliurus* individuals, no significant trend was explored. To further study, the triterpenoids PCA plot was combined with genotypic subgroup data, and it was found that almost all the high-triterpenoids type germplasms were in genotype subgroup 2, but the genotype subgroup 1 showed much lower content of triterpenoids than the others (Figure 4). Despite most individuals, more samples in subgroup 3 showed relatively lower high triterpenoids content than genotype subgroup 2. And this may imply that genotype subgroup clustering influenced *C. paliurus* leaf triterpenoids accumulation to some extent.

To further analyze the association between genotype subgroups and triterpenoids, a Kruskal–Wallis test was made between seven triterpenoid indicators and two genotype subgroups (Table 7), and genotype subgroup 1 was not evaluated because of the small sample size. According to the Kruskal–Wallis test result, it was found that five triterpenoid indicators exhibit a significant difference between genotype group 2 and genotype group 3 at *P* < 0.01 level. Content of a total of six triterpenoids, pterocaryoside B, pterocaryoside A, hederagenin, and oleanolic acid all showed a significant difference between genotype groups, while arjunolic acid and cyclocaric acid B were not influenced between genotype subgroup 2 and genotype subgroup 3.

**Table 7:** Kruskal–Wallis test and multiple comparisons between genotypes and triterpenoids of *C. paliurus*.

**Triterpenoids**	**Significance**	**Genotype 1**	**Genotype 2**	**Genotype 3**
Arjunolic acid	0.07	1.1 (1–1.34)Aa	3.2 (2.16–5.35)Bb	2.98 (2–4.23)Bb
Cyclocaric acid B	0.08	0.41 (0.3–0.51)Aa	1.49 (0.88–2.14)Bb	1.24 (0.79–1.86)Bb
Pterocaryoside B	0	0.44 (0.25–0.7)Aa	3.35 (1.78–5.7)Cc	1.68 (0.19–3.51)Bb
Pterocaryoside A	0	0.57 (0.25–0.73)Aa	5.01 (3.42–7.44)Cc	2.65 (1.51–4.06)Bb
Hederagenin	0	1 (0.7–1.36)ABab	1.18 (0.73–1.63)Bb	0.73 (0.49–1.13)Aa
Oleanolic acid	0	0.64 (0.51–0.68)Bb	0.44 (0.34–0.62)Bb	0.3 (0.2–0.42)Aa
Content of total six triterpenoids	0	4.18 (3.63–4.81)Aa	15 (11.27–22.24)Cc	10.34 (6.97–14.99)Bb

*Lower case letters indicate that the triterpenoid individual has a significant difference at the P < 0.05 level in genotypes; upper case letters indicate that the triterpenoid individuals of different genotypes have a significant difference at the P < 0.01 level*.

For further analysis, multiple comparisons were carried out to deeply identify the triterpenoids variation pattern (Table 7). Genotype subgroup 2 has shown the highest total triterpenoid content with the median being 15 mg/g and the quartile being 11.27–22.24 mg/g, significantly higher (*P* < 0.01) than genotype subgroup 3 with the median being 10.34 mg/g and the quartile being 6.97–14.99 mg/g. And genotype subgroup 1 has shown the lowest total triterpenoid content with the median being only 4.18 mg/g and the quartile being 3.63–4.81 mg/g. Besides, pterocaryoside B (3.35, 1.78–5.7 mg/g) and pterocaryoside A (5.01, 3.42–7.44 mg/g) of genotype subgroup 2 showed higher content than genotype subgroup 3 in *P* < 0.01 level (pterocaryoside B, 1.68 mg/g, 0.19–3.51 mg/g; pterocaryoside A, 2.65 mg/g, 1.51–4.06 mg/g). And genotype subgroup 1 has also showed the lowest pterocaryoside B (0.44, 0.25–0.7 mg/g) and pterocaryoside A (0.57, 0.25–0.73 mg/g). For hederagenin, genotype subgroup 2 showed significant higher content (1.18, 0.73–1.63 mg/g) than genotype subgroup 3 (0.73, 0.49–1.13 mg/g) at *P* < 0.01 level. And hederagenin content of genotype subgroup 1 showed no significant difference from the other two. To the arjunolic acid content, genotype subgroup 2 (3.2, 2.16–5.35 mg/g) and genotype subgroup 3 (2.98, 2–4.23 mg/g) showed no significant difference, while significant higher than genotype subgroup 1 (0.41, 0.3–0.51 mg/g) in *P* < 0.01 level. In general, most triterpenoid individuals, including total triterpenoid content, have been certified significantly correlated with the genotype differentiation of *C. paliurus* germplasm.

## Discussion

In a previous study on the genetic diversity of *C. paliurus* resources, no significant differences in coefficients were detected in a UPGMA (Li X. C. et al., 2017). In that study, the 26 *C. paliurus* populations were divided into subgroups based on genetic diversity detected using nine ISSR primers and six SSR primers (Li X. C. et al., 2017). Despite long-term geographical isolation, the 26 populations were separated into five groups at the 0.91 level in the ISSR and SSR marker-based UPGMA dendrogram. However, the UPGMA method seemed to produce ambiguous results that were rather limited for association analyses of population division. In this study, instead of conducting analyses at the population or individual levels as was done in previous studies (Cao et al., 2018; Liu et al., 2018; Deng et al., 2019; Zhou et al., 2019), we conducted a PCA to analyze the phylogenetic relationships in the *C. paliurus* population. The 26 populations were divided into three clear subgroups according to the first two components, this partly agreed with the results of the previous UPGMA clustering analysis (Li X. C. et al., 2017), but the groups were more clearly defined for further analyses.

The genotype × environment interaction is considered to play an important role in secondary metabolite accumulation (Deng et al., 2019). In this research, it was found that genotype subgroups influenced secondary metabolite accumulation, consistent with the results of Deng et al. (2015, 2017). Deepak et al. (2018) found that genotype had a stronger effect than provenance on variations in secondary metabolite accumulation in leaves of silver birch. Genotypes also have been shown to differ in their responses to environmental factors (Keinänen et al., 1999; Laitinen et al., 2005). Accordingly, the results of this research showed that genotype generally had a stronger effect than environmental factors on triterpenoids differentiation in leaves of *C. paliurus*.

In a previous study, environmental factors have been considered to be the main reason for influencing plant triterpenoids accumulation. Yeh et al. (2016) found that nitrogen significantly promoted triterpenoid production in an endophytic fungus. Park et al. (2005) and Schlag and McIntosh (2006) found that the triterpenoid contents and types differed among the same plant species grown in different environments. Sun et al. (2018, 2019) and Fan et al. (2013) found that *Sapindus mukorossi* growing at sites with higher temperatures had higher contents of the triterpenoid saponin in fruits. While fewer researches have focused on triterpenoid differentiation between genotypes. Deng et al. (2018) concluded that the environment had a stronger influence than the genotype on total triterpenoid content, but the genotype had a stronger influence than the environment on the accumulation of individual triterpenoids.

Our previous research has considered that altitude and longitude were significantly correlated with *C. paliurus* triterpenoid (Sun et al., 2021), while the conclusion did not take the genotype influence into account. In this research, according to the statistical results, total triterpenoids have shown a significant difference between genotypes, which can improve insulin resistance, improve glucolipid metabolism disorder, and have the effects of lowering blood sugar and blood lipid. Besides, the response of different triterpenoid individuals to genotype differences was not consistent. Tetracyclic triterpenes and pentacyclic triterpenes have the same synthetic precursors, but correspond to different synthetic paths (Deng et al., 2017). Therefore, the response of these two different types of triterpenoids to genotype differentiation may also be different, which is agreed with Deng et al. (2018). In this research, arjunolic acid, cyclocaric acid B, hederagenin, and oleanolic acid were pentacyclic triterpenoid acids, while pterocaryoside B and pterocaryoside A were tetracyclic triterpenoid saponins, which exhibit a significant difference between genotype subgroups. Pterocaryoside B and pterocaryoside A are both specific to the species and are highly sweet secodammarane glycosides (Kennelly et al., 1995; Kinghorn and Soejarto, 2002). The genotype difference in pterocaryoside B and pterocaryoside A accumulation may lead to different sweetness and other potential bioactivities.

More previous studies have investigated genotypic variation at population or family levels, and the conclusions are drawn only apply to those specific populations or families. However, in this study, we detected a general pattern of triterpenoids accumulation among *C. paliurus* resources and found that triterpenoid accumulation responses differed among genotype subgroups. According to the genotype subgroups defined based on molecular marker data, the *C. paliurus* germplasm resources could be divided into a high-triterpenoid group and others. Previous studies have suggested that environmental factors have stronger effects than the genetic background on the accumulation of triterpenoids (Deng et al., 2018) in the leaves of *C. paliurus*. However, the wide variations in secondary metabolite contents detected in this study implied that genotype may also be an important factor. Therefore, the extent to which the variations among populations were influenced by genotype and environmental factors was evaluated.

## Conclusion

In this study, three genotype subgroups exhibit significant distributing characteristics to the environmental gradient. Different genotype subgroups of *C. paliurus* showed different patterns of total triterpenoids accumulation. The germplasm could be divided into the high-triterpenoids group and normal groups, which will be useful for further selection. Among triterpenoids, different triterpenoid individuals' variation pattern to genotype differences was not consistent, pterocaryoside B and pterocaryoside A significantly accumulated in the high-triterpenoids genotype group. This information will be useful for the targeted breeding of *C. paliurus* for particular purposes, and for optimizing cultivation conditions to favor the accumulation of certain groups of metabolites. More secondary metabolites should be considered in further association analysis research to better understand the effect of the genotype × environment interaction on secondary metabolite accumulation in *C. paliurus* leaves.

## Data Availability Statement

The original contributions presented in the study are included in the article/supplementary material, further inquiries can be directed to the corresponding authors.

## Author Contributions

CS and SF: methodology, experimental design, and writing—review and editing. WY, HD, YC, and XL: data curation. CS: data analysis and writing—original draft preparation. XS: funding acquisition. All authors have read and agreed to the published version of the manuscript.

## Funding

This work was supported by the National Natural Science Foundation of China (31971642), the Key Research and Development Program of Jiangsu Province (BE2019388).

## Conflict of Interest

The authors declare that the research was conducted in the absence of any commercial or financial relationships that could be construed as a potential conflict of interest.

## Publisher's Note

All claims expressed in this article are solely those of the authors and do not necessarily represent those of their affiliated organizations, or those of the publisher, the editors and the reviewers. Any product that may be evaluated in this article, or claim that may be made by its manufacturer, is not guaranteed or endorsed by the publisher.

## Abbreviations

ISSR: intersimple sequence repeat
PAGE: polyacrylamide gel electrophoresis
PCA: principal component analysis
PCR: polymerase chain reaction
SSR: simple sequence repeat
UPGMA: unweighted pair group method with arithmetic mean.

## Appendix

**Table A1:** Identification of 6 triterpenoid compounds from leaves of *C. paliurus* by developed HPLC-Q-TOF-MS.

**Peak No**.	**t_R_ (min)**	**[M-H]-**	**MS/MS fragment ion (m/z)**	**Formula**	**Identification**
12	47.1	487.343	445.2942, 401.3056, 389.2698	C_30_H_48_O_5_	Arjunolic acid
13	49.7	485.328	/	C_30_H_46_O_5_	Cyclocaric acid B
14	54.2	621.400	521.3107, 489.3571	C_35_H_58_O_9_	Pterocaryoside B
15	60.0	635.416	535.3265, 489.3573	C_36_H_60_O_9_	Pterocaryoside A
16	60.5	471.348	145.0286	C_30_H_48_O_4_	Hederagenin
20	83.5	455.355	/	C_30_H_48_O_3_	Oleanolic acid
